# Receptor Diversity and Host Interaction of Bacteriophages Infecting *Salmonella enterica* Serovar Typhimurium

**DOI:** 10.1371/journal.pone.0043392

**Published:** 2012-08-21

**Authors:** Hakdong Shin, Ju-Hoon Lee, Hyeryen Kim, Younho Choi, Sunggi Heu, Sangryeol Ryu

**Affiliations:** 1 Department of Food and Animal Biotechnology, Department of Agricultural Biotechnology and Center for Agricultural Biomaterials, Seoul National University, Seoul, Korea; 2 Department of Food Science and Biotechnology, Kyung Hee University, Yongin, Korea; 3 Microbial Safety Division, National Academy of Agricultural Science, Rural Development Administration, Suwon, Korea; University of Osnabrueck, Germany

## Abstract

**Background:**

*Salmonella enterica* subspecies *enterica* serovar Typhimurium is a Gram-negative pathogen causing salmonellosis. *Salmonella* Typhimurium-targeting bacteriophages have been proposed as an alternative biocontrol agent to antibiotics. To further understand infection and interaction mechanisms between the host strains and the bacteriophages, the receptor diversity of these phages needs to be elucidated.

**Methodology/Principal Findings:**

Twenty-five *Salmonella* phages were isolated and their receptors were identified by screening a Tn5 random mutant library of S. Typhimurium SL1344. Among them, three types of receptors were identified flagella (11 phages), vitamin B_12_ uptake outer membrane protein, BtuB (7 phages) and lipopolysaccharide-related O-antigen (7 phages). TEM observation revealed that the phages using flagella (group F) or BtuB (group B) as a receptor belong to *Siphoviridae* family, and the phages using O-antigen of LPS as a receptor (group L) belong to *Podoviridae* family. Interestingly, while some of group F phages (F-I) target FliC host receptor, others (F-II) target both FliC and FljB receptors, suggesting that two subgroups are present in group F phages. Cross-resistance assay of group B and L revealed that group L phages could not infect group B phage-resistant strains and reversely group B phages could not infect group L SPN9TCW-resistant strain.

**Conclusions/Significance:**

In this report, three receptor groups of 25 newly isolated *S.* Typhimurium-targeting phages were determined. Among them, two subgroups of group F phages interact with their host receptors in different manner. In addition, the host receptors of group B or group L SPN9TCW phages hinder other group phage infection, probably due to interaction between receptors of their groups. This study provides novel insights into phage-host receptor interaction for *Salmonella* phages and will inform development of optimal phage therapy for protection against *Salmonella*.

## Introduction

Emergence of antibiotic-resistant pathogens due to abuse of various antibiotics is driving the development of alternative approaches to pathogen control. Bacteriophages are considered a possible alternative biocontrol agent for bacterial pathogens [1], [2]. This approach has advantages including narrow species specificity and safety for human applications [3]. As an example, a clinical trial in which volunteers were given oral doses of T4 phage indicated that it was safe [4]. In addition, a cocktail (ListShield™, Intralytix, Inc., Baltimore, MD, USA) of *Listeria-*infecting bacteriophages was approved by the US Food and Drug Administration in 2006, gaining the status of “Generally Recognized as Safe” [2], [5], supporting that bacteriophage may be a good candidate as a biocontrol agent for human applications.

*Salmonella* is a Gram-negative foodborne pathogen causing 1.4 million cases of salmonellosis including 17,000 hospitalization and 600 deaths every year in US [6], [7]. *S. enterica* serovar Typhimurium is common serotype in human infection and is frequently isolated from clinical and non-clinical samples from chicken sources. A large proportion of *S*. Typhimurium strains are resistant to several antimicrobial drugs, for example the multi-drug resistant *S.* Typhimurium phage type DT104 [8]. Therefore, use of *S*. Typhimurium bacteriophages is now getting more attractive as an alternative approach in the treatment for antibiotic-resistant pathogens.

Recent reports have described the isolation of new *Salmonella* bacteriophages and evaluation of their bactericidal effects [9]–[12]. For example, *Salmonella*-specific phage st104a or st104b reduced the bacterial cell number by up to 2 logs within 1 h of each phage infection [12]. Φ25 phage reduced *S.* Typhimurium viable cell number up to 2.19 logs within 24 h [13], [14]. However, the rapid emergence of phage-resistant *Salmonella* is an obstacle to effective biocontrol using phages [5], [14]–[17]. To reduce the impact of phage-resistance, phage cocktails have been developed and found to be effective in control of phage-resistant *Salmonella* [15], [18]–[20]. For example, a phage cocktail containing 45 different phages reduced *Salmonella* cell numbers up to 5 log in 2 h [15]. The phage cocktail approach can also broaden the host range, for example, one phage cocktail designed for serovar Typhimurium increased the host range to include *S. enterica* serovar Enteritidis and serovar Kentucky [21].

Because attachment of bacteriophage to the specific receptor of the host bacteria is the critical first step of phage infection [22], mutation of the receptor is the most frequent route to phage-resistance of the host. To date, several *Salmonella* phage receptors are known including FhuA [23], TolC [24], BtuB [25], [26], OmpC [27], Vi capsular antigen [28], lipopolysaccharide (LPS) [29], and flagella [13]. The study of phage receptors is expected to provide insight into the emergence of phage-resistance in *Salmonella* and guide optimization of phage cocktails for *Salmonella* control.

In this study, host receptors for 25 new *Salmonella* phages were determined using a phage-resistant Tn*5*-insertion random mutant library of *S.* Typhimurium. Cross infection studies with these phages and resistant strains revealed novel phage-host interactions and infection mechanisms. Further investigation of diversity of host receptors in *Salmonella* phages will increase our ability to circumvent phage resistance using phage cocktails and thus prevent food spoilage due to *S*. Typhimurium.

## Materials and Methods

### Bacterial Strains and Growth Conditions

Bacterial strains used in this study are listed in Table 1. All strains were grown in Luria-Bertani (LB) broth medium (Difco, Detroit, MI, USA) at 37°C with shaking for 12 h. *Salmonella enterica* serovar Typhimurium strain SL1344 was used for isolation of *Salmonella-*infecting phages from the collected samples, and prophage-free *S.* Typhimurium strain LT2C [30] (Cancer Research Center, Colombia, MO, USA) was used for purification of phages.

**Table 1:** Host range of bacteriophages isolated.

Host	Lytic spectrumsa,b												References^d^
	GroupF-I			GroupF-II		Group B					Group L		
	I	II	III	IV	V	VI	VII	VIII	IX	X	XI	XII	
*Salmonella enterica*													
subsp. *enterica* serovar Typhimurium SL1344	T	T	T	T	T	C	C	C	C	C	T	C	NCTC
subsp. *enterica* serovar Typhimurium UK1, LT2, LT2C^c^*	T	T	T	T	T	C	C	C	C	C	T	T	[66], [67], [30]
subsp. *enterica* serovar Typhimurium ATCC 14028	–	–	–	T	I	T	T	T	T	T	T	T	ATCC
subsp. *enterica* serovar Typhimurium ATCC 19586	C	T	C	T	T	C	C	C	C	T	T	C	ATCC
subsp. *enterica* serovar Typhimurium ATCC 43147	T	T	T	T	T	–	I	–	–	I	T	C	ATCC
subsp. *enterica* serovar Typhimurium DT104	T	T	T	T	T	–	–	–	–	–	T	C	[68]
subsp. *enterica* serovar Enteritidis ATCC 13078	–	–	–	–	–	C	C	T	T	C	T	C	ATCC
subsp. *enterica* serovar Typhi Ty 2-b	–	–	–	–	–	–	T	–	–	T	–	–	IVI
subsp. *enterica* serovar Paratyphi A IB 211	T	–	–	–	T	C	C	C	C	C	–	–	IVI
subsp. *enterica* serovar Paratyphi B IB 231	–	–	–	–	–	T	T	T	T	T	T	C	IVI
subsp. *enterica* serovar Paratyphi C IB 216	–	–	–	–	–	T	T	T	T	T	T	–	IVI
subsp. *enterica* serovar Dublin IB 2973	–	–	–	–	–	–	T	T	T	C	–	–	IVI
subsp. *arizonae* ATCC 13314	–	T	T	T	T	–	–	–	–	–	–	–	ATCC
subsp. *arizonae* ATCC 12324	–	T	T	T	T	C	C	C	C	C	–	–	ATCC
subsp. *salamae* ATCC 15793	T	T	T	T	T	–	–	–	–	–	–	–	ATCC
subsp. *salamae* ATCC 43972	–	–	–	–	–	–	–	–	–	C	–	–	ATCC
subsp. *indica* ATCC 43976	–	–	–	–	–	T	C	T	T	C	–	–	ATCC
subsp. *houtenae* ATCC 43974	–	–	–	–	–	–	–	–	–	–	–	–	ATCC
subsp. *diarizonae* ATCC 43973	–	–	–	–	–	T	T	T	C	T	–	–	ATCC
*E. coli*													
K-12, DH5α, DH10B	–	–	–	–	–	C	C	C	C	C	–	–	[69], ATCC, [70]
O157:H7 ATCC 43888	–	–	–	–	–	–	–	C	–	–	–	–	ATCC
O157:H7 ATCC 43895	–	–	–	–	–	–	–	T	–	–	–	–	ATCC
Gram-negative bacteria													
*Shigella flexineri* 2a strain 2457T	–	–	–	–	–	C	–	C	C	–	–	–	IVI
*Shigella boydii* IB 2474	–	–	–	–	–	–	–	–	–	–	–	–	IVI
*Vibrio fischeri* ES-114 ATCC 700601	–	–	–	–	–	–	–	–	–	–	–	–	ATCC
*Pseudomonas aeruginosa* ATCC 27853	–	–	–	–	–	–	–	–	–	–	–	–	ATCC
*Cronobacter sakazakii* ATCC 29544	–	–	–	–	–	–	–	–	–	–	–	–	ATCC
Gram-positive bacteria													
*Enterococcus faecalis* ATCC 29212	–	–	–	–	–	–	–	–	–	–	–	–	ATCC
*Staphylococcus aureus* ATCC 29213	–	–	–	–	–	–	–	–	–	–	–	–	ATCC
*Bacillus cereus* ATCC 14579	–	–	–	–	–	–	–	–	–	–	–	–	ATCC
*Listeria monocytogenes* ATCC 19114	–	–	–	–	–	–	–	–	–	–	–	–	ATCC

a Lytic spectrum: I contains SPN2T, SPN3C, and SPN13B; II contains SPN8T and SPN9T; III contains SPN11T, SPN16C; IV, SPN4S, and SPN19; V contains SPN5T and SPN6T; VI contains SPN7C and SPN9C, VII, SPN10H; VIII contains SPN12C and SPN17T; IX, SPN14; X, SPN18; XI contains SPN1S, SPN2TCW, SPN4B, SPN6TCW, SPN8TCW, and SPN13U; XII, SPN9TCW.

b C, clear plaque; T, turbid plaque; I, inhibition zone; –, no infection.

c Prophage-cured strain of S. Typhimurium LT2.

d NCTC, National Collection of Type Cultures; ATCC, American Type Culture Collection; IVI, International Vaccine Institute.

### Bacteriophage Isolation and Propagation

Bacteriophages isolated in this study were listed in Table 2. No live chickens were used in this study. Seventy-six samples obtained from chicken feces collected from farms and commercially processed broiler skins obtained from markets were used as sources for isolation of *Salmonella*-specific bacteriophages. The chicken feces were collected with permissions from the farm owners and the samples were collected for the purpose of this research only. Twenty-five grams of each sample were mixed with 225 ml of sodium chloride–magnesium sulfate (SM) buffer (100 mM NaCl, 10 mM MgSO_4_·7H_2_O and 50 mM Tris·HCl, pH 7.5) without gelatin in sterile bags. Twenty-five milliliters of each homogenized sample were mixed with 25 ml of 2× concentrated LB broth and incubated with shaking at 37°C for 12 h. After centrifugation (5,000×*g* for 10 min), the supernatant was filtered using 0.22-µm pore size filters (Millipore, Billerica, MA, USA). Ten milliliters of each filtered sample were mixed with 50 ml LB broth with 10^7^ CFU/ml of overnight cultured *S.* Typhimurium SL1344. The mixture was incubated with shaking at 37°C for 12 h. After centrifugation (5,000×*g* for 10 min), the supernatant was filtrated using 0.22 µm pore size filters. The presence of phages was assessed using a plaque forming assay with molten 0.4% LB soft agar containing 10^7 ^CFU/ml of *S.* Typhimurium SL1344. After incubation at 37°C for 12 h, individual plaques were picked and eluted with 1 ml of SM buffer without gelatin. Plaque isolation and elution were repeated more than five times for pure isolation of individual phages in *S.* Typhimurium LT2C. One liter of exponentially growing *S.* Typhimurium LT2C (OD_600 nm_ = 1.0) was infected with each SPN phage at a multiplicity of infection (MOI) of approximately 1 and incubated with shaking at 37°C for 4 h. Cell debris was removed by centrifugation at 5,000×*g* for 10 min and filtered using 0.22-µm pore size filters. Phage particles were precipitated from the filtrate by addition of 10% polyethylene glycol 6,000 (Sigma, St. Louis, MO, USA). Finally, a stepped CsCl density ultracentrifugation (himac CP 100β, Hitachi, Japan) with step densities of 1.3, 1.45, 1.5, and 1.7 g/ml at 78,500×*g* for 2 h was conducted at 4°C. The bands of viral particles were withdrawn from the tube with a syringe and dialyzed using 1 L of SM buffer for 1 h and stored at 4°C.

**Table 2:** Characteristics of the isolated *S*. Typhimurium-specific bacteriophages and their identified receptors.

Group^a^ (Family)	Phage	Source	Mutated genes of the phage-resistant strains
F-I (*Siphoviridae*)	SPN2T	Chicken feces1	*flgK, fliR* b or *fliC*
	SPN3C	Chicken feces2	*flgK, fliR* or *fliC*
	SPN8T	Processed broiler skin1	*flgK, fliR* or *fliC*
	SPN9T	Processed broiler skin2	*flgK, fliR* or *fliC*
	SPN11T	Soil1	*flgK, fliR* or *fliC*
	SPN13B	Water1	*flgK, fliR* or *fliC*
	SPN16C	Chicken feces3	*flgK, fliR* or *fliC*
F-II (*Siphoviridae*)	SPN4S	Processed broiler skin3	*flgK* or *fliR*
	SPN5T	Processed broiler skin4	*flgK* or *fliR*
	SPN6T	Processed broiler skin5	*flgK* or *fliR*
	SPN19	Processed broiler skin6	*flgK* or *fliR*
B (*Siphoviridae*)	SPN7C	Processed broiler skin7	*btuB* c
	SPN9C	Processed broiler skin2	*btuB*
	SPN10H	Soil2	*btuB*
	SPN12C	Soil3	*btuB*
	SPN14	Soil4	*btuB*
	SPN17T	Silky fowl feces	*btuB*
	SPN18	Processed broiler skin8	*btuB*
L (*Podoviridae*)	SPN1S	Water2	*rfaL* d or *rfbG*
	SPN2TCW	Chicken feces1	*rfaL* or *rfbG*
	SPN4B	Processed broiler skin3	*rfaL* or *rfbG*
	SPN6TCW	Processed broiler skin5	*rfaL* or *rfbG*
	SPN8TCW	Processed broiler skin1	*rfaL* or *rfbG*
	SPN13U	Water3	*rfaL* or *rfbG*
	SPN9TCW	Processed broiler skin2	*rfaL* or *rfbG*

a F-I and F-II, flagella-specific phage group; B, BtuB-specific phage group; L, LPS-specific phage group.

b *flgK*, *fliR* mutations were complemented using pACYC184 vector expressing the *flgK* ^+^ or *fliR* ^+^ gene.

c *btuB* mutation was complemented using pACYC184 vector expressing the *btuB* ^+^ gene.

d *rfaL* mutation was complemented using pUHE21-*lacI* ^q^ vector expressing the *rfaL* ^+^ gene.

### Transposon Mutagenesis and Receptor Screening

Random insertion mutagenesis of *S.* Typhimurium SL1344 was performed using the EZ-Tn5™ <R6Kγori/KAN-2>Tnp Transposome kit (Epicentre, Madison, WI, USA) according to manufacturer’s procedure. The transposon construct was obtained by treating pMOD3 with PvuII restriction enzyme, and gel purifying the fragment from a 1% agarose gel using the QIAquick Gel Extraction Kit (Qiagen, Valencia, CA, USA). Electrocompetent cells were prepared as follows: Fifty milliliter of LB broth inoculated at 1% with an overnight culture of *S.* Typhimurium SL1344 was incubated with shaking at 37°C for 1.5 h. Cells were then harvested by centrifugation at 5,000×*g* for 10 min, and the pellet was washed three times with 1 ml ice-cold molecular grade water and resuspended with 100 µl ice-cold molecular grade water. The complexes were electroporated into *S.* Typhimurium SL1344 using the Gene-Pulser Xcell system (Bio-Rad, Hercules, CA, USA) at 2.45 kV, 200 Ω and 25 µF in 2 mm electroporation cuvettes. Transformants were selected on LB agar plates containing kanamycin (Kan, 50 µg/ml), and individual colonies were cultured and stored in LB broth with Kan (50 µg/ml) containing 15% glycerol at −80°C.

To screen mutant strains resistant to each SPN phage, the mutant library was duplicated in the 96-well plates containing LB broth supplemented with 50 µg/ml of Kan. The SPN phage was added to one of those duplicated 96-well plates (MOI = 10) after shaking at 37°C for 1.5 h and growth inhibition was compared with the corresponding 96-well plate not inoculated with phage. All plates were incubated at 37°C for an additional 3 h. Phage resistance mutants were identified by comparing the optical density at 600 nm of the plates with and without phage using an iMark microplate absorbance reader (Bio-Rad). Rescue cloning of transposed genomic DNA and partial sequencing was conducted to identify the Tn*5* transposon insertion sites according to the manufacturer’s protocol.

### Construction of Deletion Mutants and Complementation Plasmids

*S.* Typhimurium SL1344 derivatives with deletions of *flgK*, *fliR*, *fliC*, *fljB, fliC/fljB* or *rfaL* genes were constructed using the lambda red recombination method [31]. The kanamycin resistance (Kan^R^) cassette from plasmid pKD13 was amplified using primers specific for each gene, for example, *flgK*-lamb-F1 and *flgK* -lamb-R1 for *flgK.* Sequences of all primers for these constructs are provided in Table S1. The polymerase chain reaction (PCR) products were transformed into *S.* Typhimurium SL1344 harboring pKD46 and integrated into the chromosomal *flgK*, *fliR*, *fliC*, *fljB* or *rfaL* genes. Finally, the Kan^R^ cassette was removed using pCP20 plasmid following the procedure of Cherepanov et al. [32]. For construction of SL1344 Δ*fliC* Δ*fljB*, phage P22-mediated transductions were performed as described previously [19]. For complementation of the *flgK*, *fliR* or *rfaL* deletion mutations, the *flgK*, *fliR* and *rfaL* genes were amplified from *S.* Typhimurium SL1344 using the primers *flgK*-pACYC-F and *flgK*-pACYC-R for *flgK, fliR*-pACYC-F and *fliR*-pACYC-R for *fliR*, *rfaL*-pUHE-F and *rfaL*-pUHE-R for *rfaL*. The PCR products were digested with BamHI and EcoRI and ligated into BamHI/EcoRI-digested pACYC184 [33] or pUHE21-*lacI* ^q^ expression vector [34]. Plasmid constructs were confirmed by sequencing, and then transformed into deletion mutant strains selecting for ampicillin (Amp)-resistant transformants on LB with 50 µg/ml of Amp. Transformants were confirmed by colony PCR and susceptibility to SPN phages was tested.

### Electron Microscopy

The morphology of CsCl-purified SPN phages was determined using transmission electron microscopy (TEM). Concentrated viral samples were diluted with SM buffer without gelatin and 5 µl of each phage sample was applied to the surface of carbon coated copper grids. Excess volume was removed by carefully touching the side of grid with filter paper and 5 µl 2% uranyl acetate (pH 4.0) was spotted on the grid for negative staining and removed after a short interval. The prepared samples were observed using TEM (LIBRA 120, Carl Zeiss, Switzerland) at 80 kV. Taxonomy of the SPN phages was determined according to the guidelines of the International Committee on Taxonomy of Viruses [35].

### Host Range Determination by Spotting Assay

A 100-µl aliquot of bacterial culture was added to 6 ml molten 0.4% LB agar and then poured on a 1.5% LB agar plate. After solidification of the top agar, 10 µl serially diluted phage suspension ranging from 10^2^ to 10^5^ plaque forming unit/ml was spotted on the top agar and the plates were incubated at 37°C for 12 h. After incubation, the sensitivity of indicator strains to the tested phages was determined by degrees of clearing in the spots or plaques (Table 1). The plaque assay was performed in triplicate.

### Isolation of Phage-resistant Strains and Determination of Cross Resistance

To investigate influence of resistance against one type of receptor to infection by phages using different receptors, resistance strains were developed against phages and then they were used for the infections by phages using different host receptors. Group L phage-resistant strains showing resistance to re-infection were isolated using high-titer overlay assay following modified Kim and Ryu’s protocol [26]. In this modified protocol, separate colonies of group L phage-resistant strains were obtained by additional streaking on LB agar plate. Because high-titer overlay assay did not work for isolation of transiently phage-resistant strains by group F, group B, and group L SPN9TCW phage infections, they were isolated using high-titer broth assay to increase the yield of the phage-resistant strains and to maintain resistance in the presence of phages. For the high-titer broth assay, phages were added to an LB broth culture of *S.* Typhimurium LT2C (OD_600 nm_ = 1.0) at MOI = 100, and the culture was incubated with shaking at 37°C until the OD_600 nm_ reached 1.0, again. To remove excessive phages, the phage-infected cells were then harvested by centrifugation at 5,000×*g* for 10 min, resuspended in 200 µl ice-cold molecular grade water and used as a host for the second infection of SPN phages using different host receptors. The host resistance to the second infection was monitored using plaquing assays as described above.

### Lysogen Induction

All phage-resistant strains of *S.* Typhimurium LT2C were cultivated at 37°C until OD_600 nm_ reached to 1.0 and 0.5 µg/ml of mitomycin C (Sigma) was added to the cultures. Then, these cultures were additionally incubated at 37°C for 2 h. After incubation, the cells were removed by centrifugation and filtration and the supernatant was collected. The spotting assay of this supernatant with *S.* Typhimurium LT2C was conducted to confirm the lysogen formation. To confirm the unstable lysogen formation of group F phage-resistant strains, the group F, B, L-SPN9TCW phage-resistant strains were plate in green plate (Evans blue uranine agar plate, 0.5% NaCl, 1% Tryptone, 0.5% Yeast extract, 0.5% K_2_HPO_4_, 0.04 M Glucose, 0.04% Evans blue, 0.04% Uranine, and 1.5% Micro agar, final concentration) as the procedure developed by Chan et al. [19].

### Real-time Reverse Transcription (RT)-PCR

Total RNA was isolated from *S.* Typhimurium using the RNeasy Mini Kit (Qiagen) and converted to cDNA using the Omniscript RT Kit (Qiagen) and random hexamers (Invitrogen, Carlsbad, CA, USA) using manufacturer’s instructions. Quantitative real-time RT-PCR was performed as previously described [36] with primers listed in Table S1.

### Sodium Dodecyl Sulfate-polyacrylamide Gel Electrophoresis (SDS-PAGE)

Flagellin of *S*. Typhimurium was isolated as previously described [37], and suspended in loading buffer (0.05 M Tris·HCl pH 8.0, 1.6% SDS, 25% glycerol, 5% 2-mercaptoethanol, 0.003% bromophenol blue, final concentration). Samples were heated in boiling water for 3 min, and then loaded in a well of a 12% Ready Gel Tris·HCl gel (Bio-Rad) in 1X Tank Buffer (0.025 M Tris, 0.192 M glycine, and 0.1% SDS, final concentration). Gel electrophoresis was performed using Mini-PROTEAN 3 (Bio-Rad) at 20 mA for 45 min. The gel was stained in a staining solution containing 0.25% Coomassie Brilliant Blue R250 and destained with a destaining solution containing 30% methanol and 10% glacial acetic acid.

## Results

### Bacteriophage Isolation

Between September and December 2009, 25 bacteriophages were newly isolated from 18 of 76 samples (23.7% phage recovery frequency). All phages in Table 2 were designated as *Salmonella* Phage Number (SPN) and specific numbers were used to indicate the sample sources and sometimes characters were used to differentiate the isolated phages from the same samples, respectively.

### Grouping of Bacteriophages Based on their Receptors

**Figure 1:**
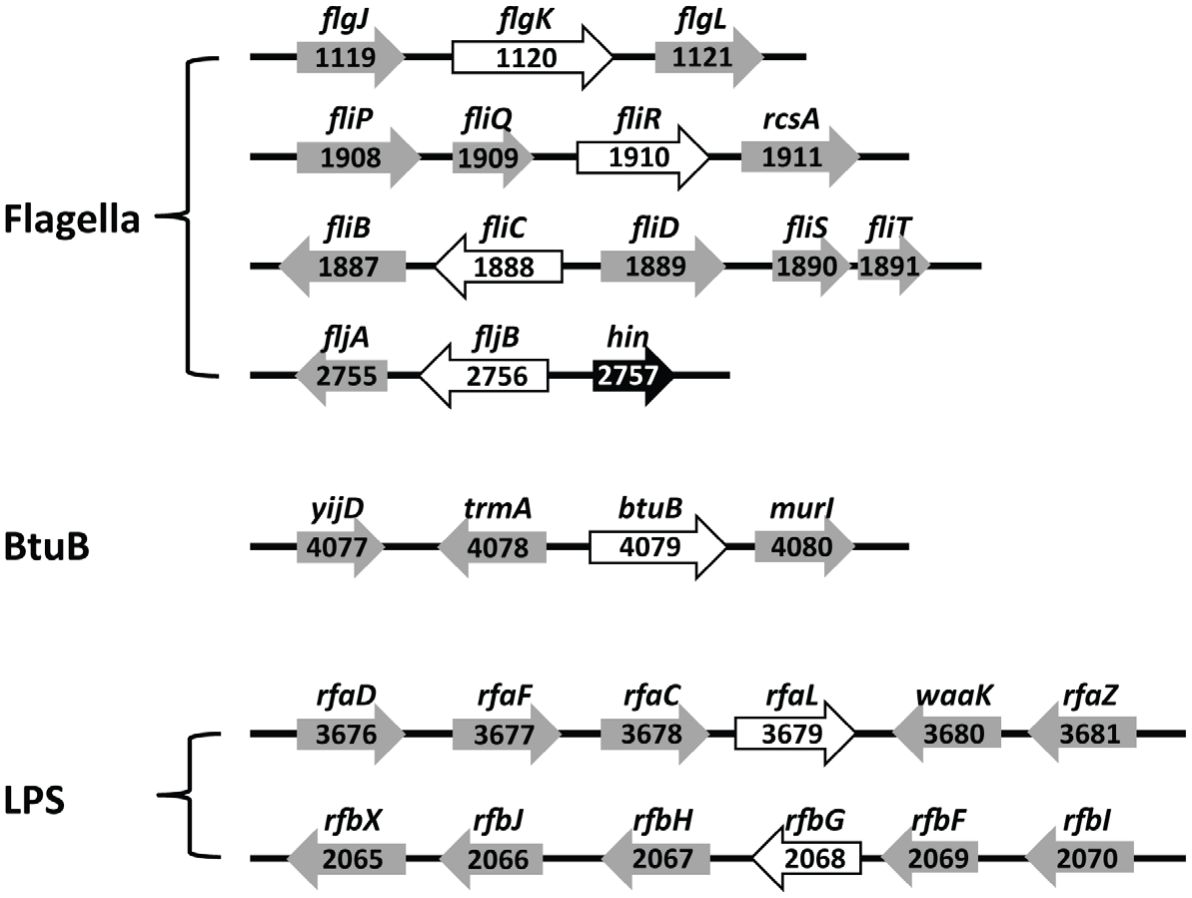
Genetic map of the receptor gene clusters and the mutated genes of resistant strains. Genes involved in the synthesis of flagella (*flgK, fliQ, fliC* and *fljB*), BtuB (*btuB*), and LPS (*rfaL* and *rfbG*) inactivated by transposon insertion were indicated by open arrows. Black arrow marked with hin is a promoter that transcribes the *fljB* gene. The numbers are locus-tag numbers indicating the locations of the genes in the genome sequence.

**Figure 2:**
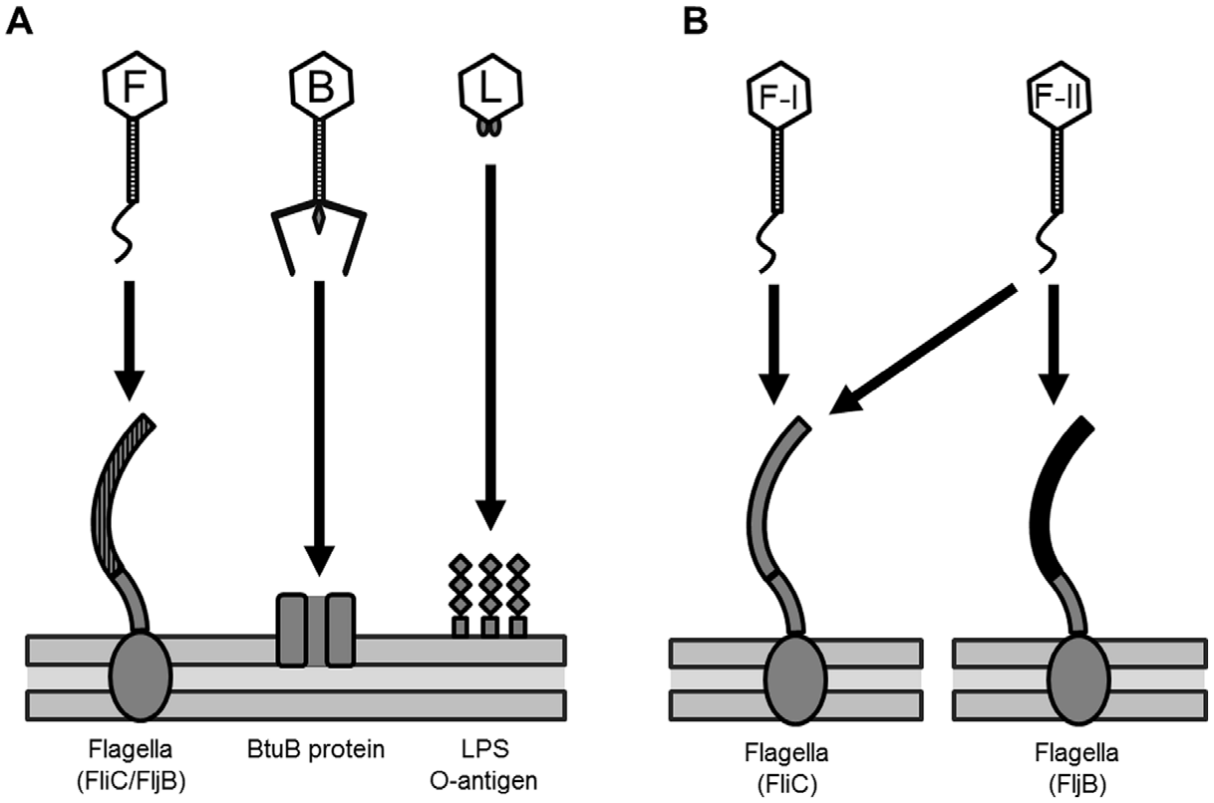
Host receptors of SPN phages. F, F-I, F-II, B, and L marked in the phage heads indicate group F, group F-I, group F-II, group B, and group L phages, respectively. (A) Group F, group B, and group L phages use flagella (FliC/FljB), BtuB, and O-antigen of LPS as host receptors, respectively. (B) Group F-I and group F-II phages use FliC (grey-colored) and FliC/FljB (black-colored) in the host flagella.

To determine the host receptors for 25 phages, a Tn5 random mutant library of *S.* Typhimurium SL1344 containing thousands of Tn5-inserted mutants was constructed and mutant strains resistant to each phage via inactivation of host receptor genes by Tn5 insertions were screened. Interestingly, sequence analysis of the Tn*5* insertions in the phage-resistant mutants revealed only three different types of genes: flagellar production genes, the gene encoding the vitamin B_12_ uptake outer membrane protein, and genes involved in LPS-related O-antigen production. The Tn5 random mutant library screening results were confirmed by targeted inactivation of the identified genes by lambda red recombination method and complementation experiments. The host receptor genes deleted by the lambda red recombination method are indicated in the receptor gene clusters presented in Fig. 1. Based on the deleted specific genes for formation of phage receptors, 25 phages were grouped into group F, group B, and group L phages, respectively (Table 2 and Fig. 2A).

The deleted genes involved in flagella production included genes encoding the flagella hook-filament junction protein (*flgK*), a putative component of the type III flagella export apparatus (*fliR*), and the gene encoding flagellin (*fliC*). Because *S*. Typhimurium expresses either one of two flagellin genes, *fliC* or *fljB* [38], we tested whether the group F phages can use both flagellins by phage infection analysis of *fliC* and *fljB* deletion mutants. The group F phages can be categorized into two groups, F-I and F-II, as shown on Table 3 and Fig. 2B. The group F-I phage could not infect the *fliC* mutant or the *fliC/fljB* double mutant, but could infect the *fljB* mutant, suggesting that the group F-I phage can only use FliC as a receptor. Group F-II phage could not infect the *fliC/fljB* double mutant but could infect the *fliC* and the *fljB* single mutants, suggesting that the group F-II phage can use either FliC or FljB as a receptor (Table 3 and Fig. 2B).

**Table 3:** Flagellin-targeting phages: receptor and sensitivity patterns based on specific gene mutation.

Strain genotype	Receptor present	Resistance profilesa,b	
		F-I phage	F-II phage
Δ*fliC*	FljB only	R	S
Δ*fljB*	FliC only	S	S
Δ*fliC* Δ*fljB*	neither	R	R

a F-I, flagella-targeting phage group I; F-II, flagella-targeting phage group II.

b S, sensitive to infection; R, resistant to infection.

One group of resistant mutants has deletion mutations in the *btuB* gene encoding the membrane transporter for vitamin B_12_, suggesting that BtuB is a group B phage receptor. The mutated genes in the O-antigen biosynthesis are O-antigen ligase (*rfaL*) and CDP glucose 4,6-dehydratase (*rfbG*). Complementation of the deleted genes with pACYC184 and pUHE21-*lac^q^* expression vectors containing the wild-type genes restored susceptibility, supporting that O-antigen is a receptor for group L phage infection. Overall, 11 out of 25 phages use flagella (group F phage), seven out of 25 use BtuB (group B phage), and another seven out of 25 use LPS-related O-antigen (group L phage) as receptors. Although the *Salmonella* outer membrane proteins (OMPs) such as TolC [24], FhuA [23] and OmpC [27] are known receptors for some phages, no phages using those receptors was present in this set of 25 phages. It is not clear why BtuB was the only OMP detected as a receptor in this study.

### Morphology

**Figure 3:**
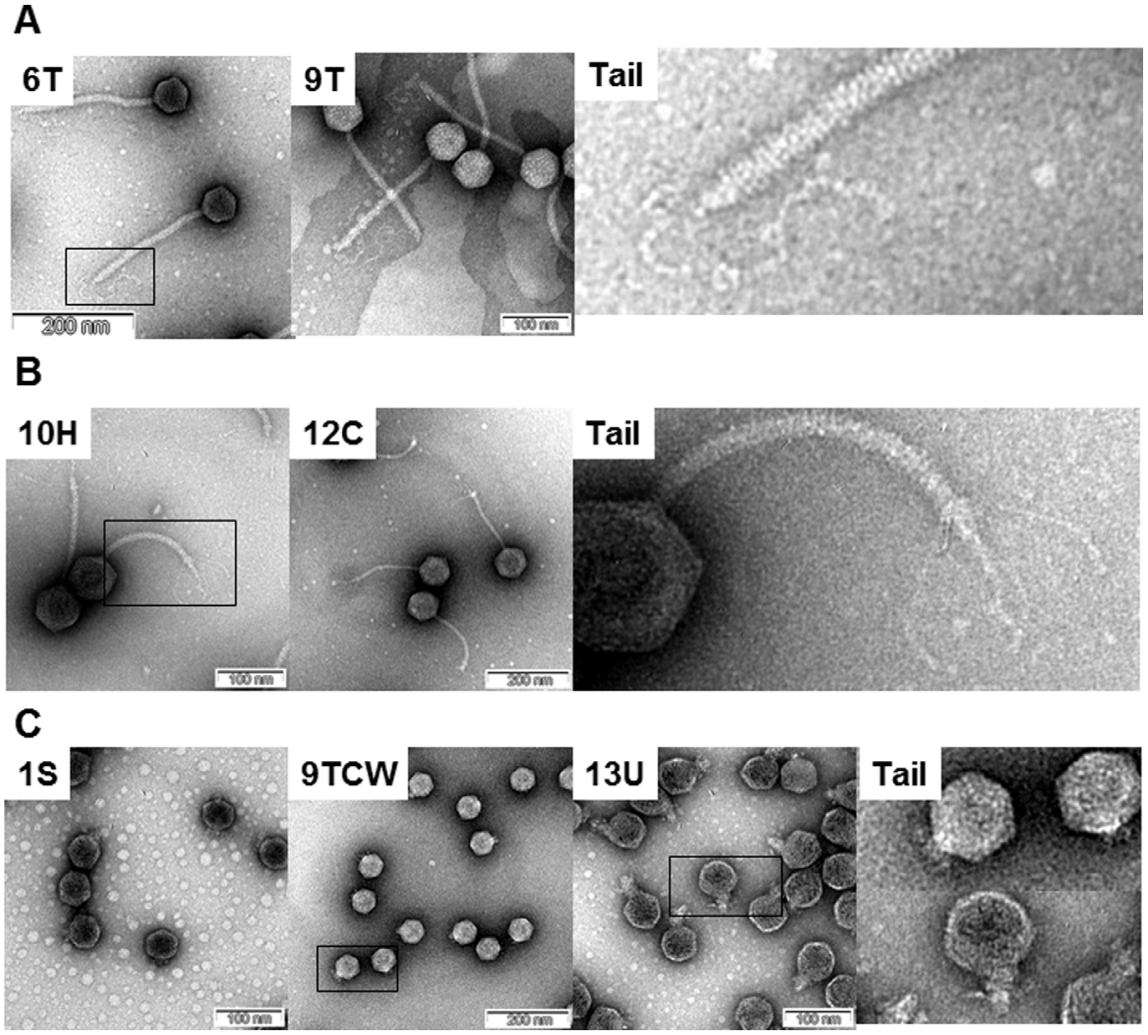
TEM morphology of representative SPN phages. Each phage name is indicated in the upper left corner of the picture. The representative tails of each group of phage were marked with boxes and the enlarged pictures were shown on the right. (A) Phages using flagella as a receptor. (B) Phages using BtuB as a receptor. (C) Phages using LPS as a receptor.

All 25 phages could be categorized into three morphological groups (Fig. 3). Interestingly, this morphological grouping is correlated with the grouping of *Salmonella*-specific phages based on their receptors (Table 2). All of the group F and B phages have isometric heads and non-contractile, cross-banded tails that are longer than the tails of the group L phages (Fig. 3). These phages can be classified into the B1 morphotype of the *Siphoviridae* family, although the group F phages have a single, long, kinky tail fiber structure and the group B phages have four or five L-shaped fibers (Fig. 1A and 1B). The group L phages are classified as members of the *Podoviridae* family. They have isometric heads with very short tails that are distinct from other groups.

### Host Range of the Isolated Bacteriophages

Infection analysis of 25 *Salmonella-*specific bacteriophages was conducted with 21 *Salmonella* strains, five *E. coli* strains and nine other species of Gram-negative and Gram-positive bacteria (summarized in Table 1). In general, SPN bacteriophages infect *S.* Typhimurium strains but not other Gram-negative and Gram-positive bacteria, suggesting that they are specific to *S.* Typhimurium. The group F phages also infect *S. enterica* subsp. *arizonae* and subsp. *salamae*, and the group B phages can infect several strains of *E. coli*, indicating that these bacteriophages have a host range that extends to other *Salmonella* subspecies or *E. coli* strains. In addition, group L phages and some group F phages infect *S.* Paratyphi. The group B phages have much broader host range than the group F and L phages. They can infect *E. coli* and *Shigella flexneri* strains as well as *Salmonella* (Table 1), suggesting that the BtuB proteins of *S.* Typhimurium, *E. coli,* and *Shigella flexneri* are similar. Comparative sequence analysis of BtuB proteins in the group B phage susceptible strains *S.* Typhimurium LT2, *E. coli* MG1655 and *Shigella flexineri* 2a strain 2457T revealed >87% identity at the amino acid level between *S*. Typhimurium and the other two species, whereas *S.* Typhimurium and *Vibrio fischeri* ES-114, which is not susceptible to group B phage, share <35% identity at the amino acid level. These findings support the hypothesis that the group B phage receptor motifs are shared among *Salmonella*, *E. coli* and *Shigella*. Comparative host range analysis of group F-I and F-II phages revealed that F-II phages infect a larger number of *S.* Typhimurium isolates (data not shown), probably because the F-II phages can use either the FliC or the FljB flagellin as a receptor while the F-I phages can only use FliC (Table 3 and Fig. 2B).

### Lysogenization

It is intriguing that while the group F and L phages generally make turbid plaques, and thus may be temperate phages, the group B phages make clear plaques and may be virulent phages [39]. Induction experiments in which mitomycin C was used to induce lytic growth indicate that the group L phage-resistant *S.* Typhimurium LT2C strains carry an inducible prophage. In contrast, mitomycin C treatment of the group L SPN9TCW phage-resistant and group B phage-resistant *Salmonella* strains did not yield phage, indicating that these phages do not make lysogens in the LT2C strain consistent with the clear plaque morphology (Table 1 and 3). Most of the group F phage-resistant *Salmonella* strains did not yield phage after treatment with mitomycin C even though they make turbid plaques. Five percent of the group F phage-resistant *Salmonella* made phage in response to mitomycin C, but they also lose resistance easily upon subculturing in the absence of the phages, suggesting the possibility of formation of unstable lysogens or pseudolysogens [40] (Table 4). To confirm if transient resistance is due to unstable lysogeny or pseudolysogeny, we conducted the green plate (Evans blue uranine agar plate) experiment (Fig. S1). In the green plate experiment, only the cells lysed by phage induction make blue colonies due to pH change. It revealed that while the resistant strains to the virulent phages in group B and group L-SPN9TCW did not show any blue colony, the resistant strains to group F phages did show small number of blue colonies (approximately 5% of all colonies) in the green plates. These results indicate that a few of the strains resistant to group F phages were lysed, suggesting that group F phages do not form stable lysogens.

**Table 4:** Cross resistance of phage-resistant strains.

Resistant strain	Phage sensitivity pattern^a^			Mitomycin C induction^b^
	Group F (Flagella)	Group B (BtuB)	Group L (LPS)	
Group F	R1	S	S	N^c^
Group B	S	R1	R1	N
Group L (SPN9TCW)	S	R1	R1	N
Group L (Other)	S	S	R2	I

a R1, transiently resistant; R2, stably resistant; S, sensitive.

b N, not induced; I, induced.

c Although most of the group F phage-resistant *Salmonella* were not induced, <5% of the resistant *Salmonella* were induced by mitomycin C.

### Cross-resistance of Phage-resistant *Salmonella* to the Different Receptor Group Phages

**Figure 4:**
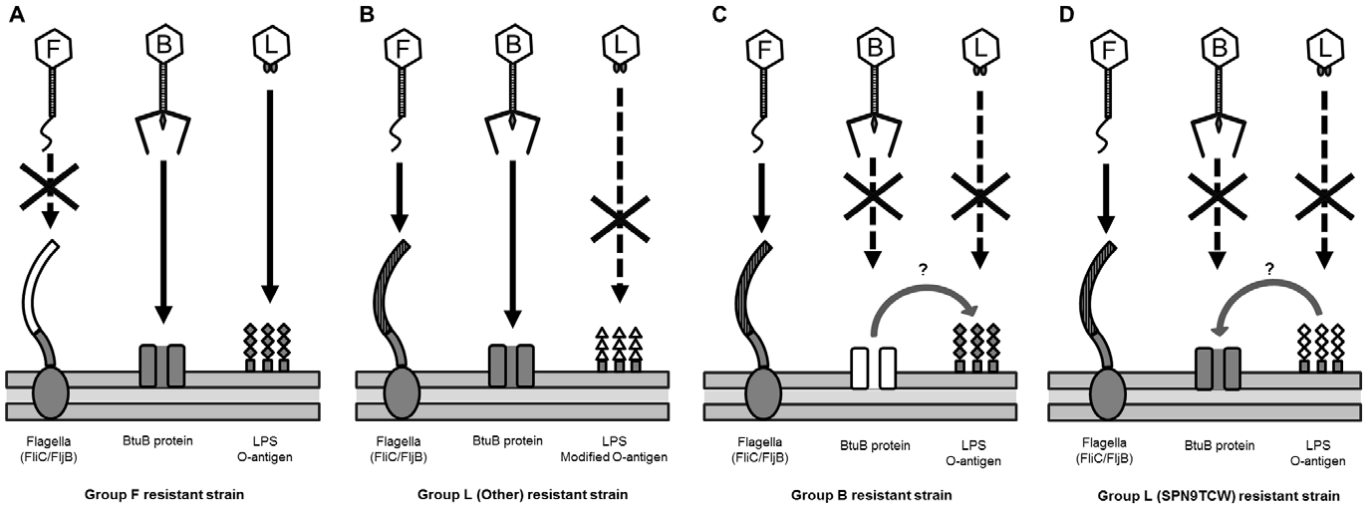
Cross-resistance of phage-resistant *Salmonella* to the different receptor group phages. F, B, and L marked in the phage heads indicate group F, group B, and group L phages, respectively. Each receptor in the phage-resistant strains is white-colored. (A) Group F phage-resistant strain is sensitive to group B and group L phages. (B) Group L phage-resistant strain is sensitive to group F and group B phages, but resistant to group L phages, due to modification of O-antigen of LPS. Modified O-antigen is indicated by white triangles. (C) Group B phage-resistant strain is sensitive to group F, but resistant to group B as well as group L phages, probably due to putative interaction between BtuB and O-antigen of LPS. (D) Group L (SPN9TCW phage)-resistant strain is sensitive to group F, but resistant to group B as well as group L phages, probably due to putative interaction between BtuB and O-antigen of LPS.

To understand the interaction of phage with the specific host receptors, derivatives of *S.* Typhimurium LT2C that are resistant to the group F, group B and group L phages described here were isolated and characterized. Group B phage-resistant *Salmonella* [26] were transiently resistant to re-infection with group B phages, and most group F phage-resistant and group L SPN9TCW phage-resistant strains also showed transient resistance to re-infection with phages from their own group. All group L phage-resistant strains, except for the strain resistant to phage SPN9TCW, showed stable phage resistance to group L phages. Interestingly, all group L phage-resistant strains except those resistant to SPN9TCW were lysogens, suggesting that the resistance for the group L phages is due to prevention of superinfection by a stable prophage (Table 4 and Fig. 4B) [41], [42]. Cross-infection of group F phages on other phage-resistant strains showed sensitivity to these phages, suggesting no mutual influence between flagellin and other phage receptors on the sensitivity to the phages (Table 4 and Fig. 4). However, cross-infection of group B phages on group L phage-resistant strains yielded two different patterns. While the group L phage-resistant strains are sensitive, the SPN9TCW phage-resistant strain is resistant to group B phage infection, depending on the formation of lysogen (Table 4 and Fig. 4BD). Furthermore, group L phages were not able to infect group B phage-resistant strains, suggesting a possible influence between the BtuB and LPS receptors on the sensitivity to the phages (Table 4 and Fig. 4C).

## Discussion

The details of the molecular interactions between phages and their host receptors that determine host specificity are not fully understood yet. Here, we isolated 25 *Salmonella*-specific phages, identified their receptors, studied their host specificity, and examined cross-resistance among the phages and phage resistant hosts. Although this study on *Salmonella* phage receptors using 25 phages does not represent the complete range of infection mechanisms used by *Salmonella-*infecting bacteriophages, the results provide novel insights into general host-phage interactions of typical *Salmonella-*infecting bacteriophages.

Bacteriophages tend to use structures exposed on the outer membrane of the host bacteria as a receptor because they are easily accessible. Unexpectedly, only three kinds of receptors were identified in this study, flagella, O-antigen, and the outer membrane protein BtuB, although several other outer membrane proteins, including FhuA, TolC, and OmpC, are characterized as phage receptors of *Salmonella*. One possible reason may be the complex nature of *Salmonella* Typhimurium LPS [43], [44], which may block access of phage to some outer membrane proteins, making it more challenging to isolate those phages. In a seeming contradiction, LPS may help attachment of group B phage to BtuB on a *S.* Typhimurium host. T5 phage, for example, has both a receptor-binding tail protein Pb5 and a L-shaped tail fiber protein on the phage tail that targets the host outer membrane protein and LPS, respectively [45], [46]. The Pb5 as a major host specificity protein is reported to mediate irreversible binding to a specific outer membrane protein and the L-shaped tail fiber protein as a helper protein provides reversible binding to LPS. Therefore, the L-shaped tail fiber protein increases the infection rate of T5 phage by stabilization of binding between phage and an outer membrane receptor via Pb5. Group B phages are in the same family as T5 phage, *Siphoviridae*, and have a similar tail structure containing an Pb5-like protein possibly targeting BtuB and an L-shaped tail fiber protein possibly targeting LPS (Fig. 3). Due to this similarity between T5 and group B phages, it is suggested that this tail fiber protein may help the binding of Pb5-like protein to BtuB to increase the infection rate of group B phages, but this proposed binding mechanism needs further study.

Morphological characterization of the group F and group L bacteriophages also provided insight into the interaction of these phages with host receptors. The group F phages have relatively long, non-contractile and cross-banded tails with a single and twisted tail fiber structure. This structure is very similar to that of chi phage that infects *E. coli, Salmonella* and *Serratia* through flagella filament receptors [47], [48], suggesting possible interaction between the group F twisted tail fibers and bacterial flagella. Group L phages are morphologically belong to *Podoviridae* and use LPS as a main receptor like other phages including ε15 [49], [50], P22 [51] and T7 [52], which all interact with LPS via major host specificity proteins. However, *Salmonella* phages of *Myoviridae* family were not isolated in this study. It is not clear why we failed to isolate phages of *Myoviridae* family. The standard phage isolation protocol employed in our study may not be suitable for isolation of *Salmonella* phages in *Myoviridae* family because the genome sizes of *Salmonella* phages of *Myoviridae* family except for *Pedovirinae* subfamily are generally bigger than those of other family phages [53]–[57]. Otherwise, there might be unknown bias in the phage isolation.

Host range analysis of group F-I phages showed that they successfully infected all *S.* Typhimurium strains tested except strain ATCC 14028. It is not clear why the group F-I phages that use only FliC as a receptor could not infect ATCC 14028 while the group F-II phages could. The complete genome sequence of strain ATCC 14028 (GenBank accession number CP001363) showed that it has a *fliC* gene and multiple sequence alignment with the *fliC* genes of susceptible *Salmonella* strains revealed no sequence differences. Real-time RT-PCR and SDS-PAGE analysis confirmed that the *fliC* gene is expressed and translated for flagella formation, indicating that FliC in the strain ATCC 14028 is functional (data not shown). The group B phages also infected all *S*. Typhimurium strains except one, strain DT104. Sequencing and real-time RT-PCR analyses of the *btuB* gene in strain DT104 showed that it is expressed (data not shown), so it is not clear why the strain is resistant. These two examples imply that there may be an additional unknown factor(s) that makes the phage and host receptor interaction more specific and complicated. However, only *S.* Typhimurium ATCC 14028 has Gifsy-3 prophage, so this prophage could cause superinfection exclusion to group F-I phages.

The phages that can make lysogen normally make turbid plaques but many other factors are involved in turbid plaque formation that it is not simple to distinguish lysogen formation based on plaque turbidity. While the lysogen generally resists superinfection by expression of the phage genes, acquisition of phage resistance by host mutation has been hardly found in lysogen.

Therefore, the aim of this lysogenization experiment was to test whether the resistance against phage infection was due to lysogenization or other factors related with a host receptor. To confirm their lysogen formation, mitomycin C was treated to induce the prophages. Group L phages except for SPN9TCW did yield phage after mitomycin C induction, substantiating the lysogen formation by most of group L phage infection. However, most of group F phage-resistant strains did not yield phage by mitomycin C induction, even though these phages make turbid plaques, suggesting that these phages may make unstable lysogen, resulting in very low frequency of mitomycin C induction (approximately 5%) in the resistant strains. It has been known that Mu-like prophages were generally not induced by mitomycin C treatment [58], suggesting the possibility that group F phage may be mitomycin C-insensitive phage. To verify this, we performed PCR detection of group F phage genomes in the resistant hosts and the green plate experiment. Recently, we sequenced completely the genomes of four phages in group F and phage-specific primers were designed. Using these phage-specific primers, PCR was conducted to detect group F phage genomes in the genomes of group F phage-resistant S. Typhimurium LT2C strains. Interestingly, very low number of group F phage-resistant strains (approximately 5%) showed the presence of group F phage genomes in the host genomes, suggesting the formation of very unstable lysogens, supporting our observation of low mitomycin C induction with group F phage-resistant strains (data not shown). And green plate experiments also showed that most of group F phage infection does not make stable lysogens. We still do not understand this very distinct feature of group F phages for unstable lysogen formation and it needs to be elucidated soon.

Analysis of cross resistance among phage-resistant strains revealed that group F phage-resistant strains are sensitive to group L or B phages (Table 4 and Fig. 4A). This sensitivity indicates that the host resistance of group F phages does not disrupt the interactions between these other phages and the cell surface receptors. In contrast, group B phage-resistant strains are resistant to their phages as well as group L phages (Table 4 and Fig. 4C). The concurrent resistance to both group B and group L phages imply that BtuB may influence the interaction between LPS and phage, as in the case of *E. coli* phage T5 [45], [46]. The interaction between LPS and phage has been reported to accelerate adsorption of phage T5 to *E. coli* even though an outer membrane protein is the cell surface receptor.

All but one group L phage-resistant strain was sensitive to infection by group B phages (Table 4 and Fig. 4B). The group L SPN9TCW phage-resistant strain was resistant to group B phages (Table 4 and Fig. 4D), even though phage SPN9TCW uses LPS as a receptor and the resistant strain does not appear to be a lysogen. Therefore stable lysogen formation of group L phages may be a key to determine the host resistance against group B phages (Table 4). Further analyses of group B phage-resistant *Salmonella* strains are required to elucidate the mechanism of cross-resistance observed against the group B and L phages.

Many *Salmonella-*specific phages that use LPS as a receptor modify LPS as a mechanism to protect from superinfection when they lysogenize a host [59], [60]. The recent complete genome sequence analysis of a lysogenic SPN1S phage in group L revealed that the phage genome encodes a GtrA and two copies of lipopolysaccharide modification acyltransferase, supporting this [61]. Even though the LPS modification protects the lysogen from a superinfection by other group L phages, the lysogen is still sensitive to phages that target other receptors such as flagella and BtuB (Table 4 and Fig. 4B). Furthermore, group F, group B and group L SPN9TCW phage-resistant strains showed transient resistance to re-infection of the same phages (Table 4). These resistant strains were collected for the cross resistant experiment after the bacterial growth resumed in the presence of the phage. In this case, these collected phage-resistant strains are probably not lysogens, suggesting that host defense mechanisms such as CRISPR [62], [63] or restriction-modification systems [64], [65] or still unknown host defense mechanisms are probably activated during growth recovery.

## Supporting Information

Figure S1 **Green plate experiment of representative phages in three phage groups.** (A) Group B SPN10H-resistant strain (B) Group L SPN9TCW-resistant strain (C) Group F SPN19-resistant strain. Red triangles indicate blue colonies on green plate.
(TIF)

Table S1 Primers used in this study.
(DOC)
