# Supplementary figures and images for: Receptor Diversity and Host Interaction of Bacteriophages Infecting Salmonella enterica Serovar Typhimurium
Source: PLoS One. 2012 Aug 21;7(8):e43392. doi: 10.1371/journal.pone.0043392 (PMC3424200; doi:10.1371/journal.pone.0043392)

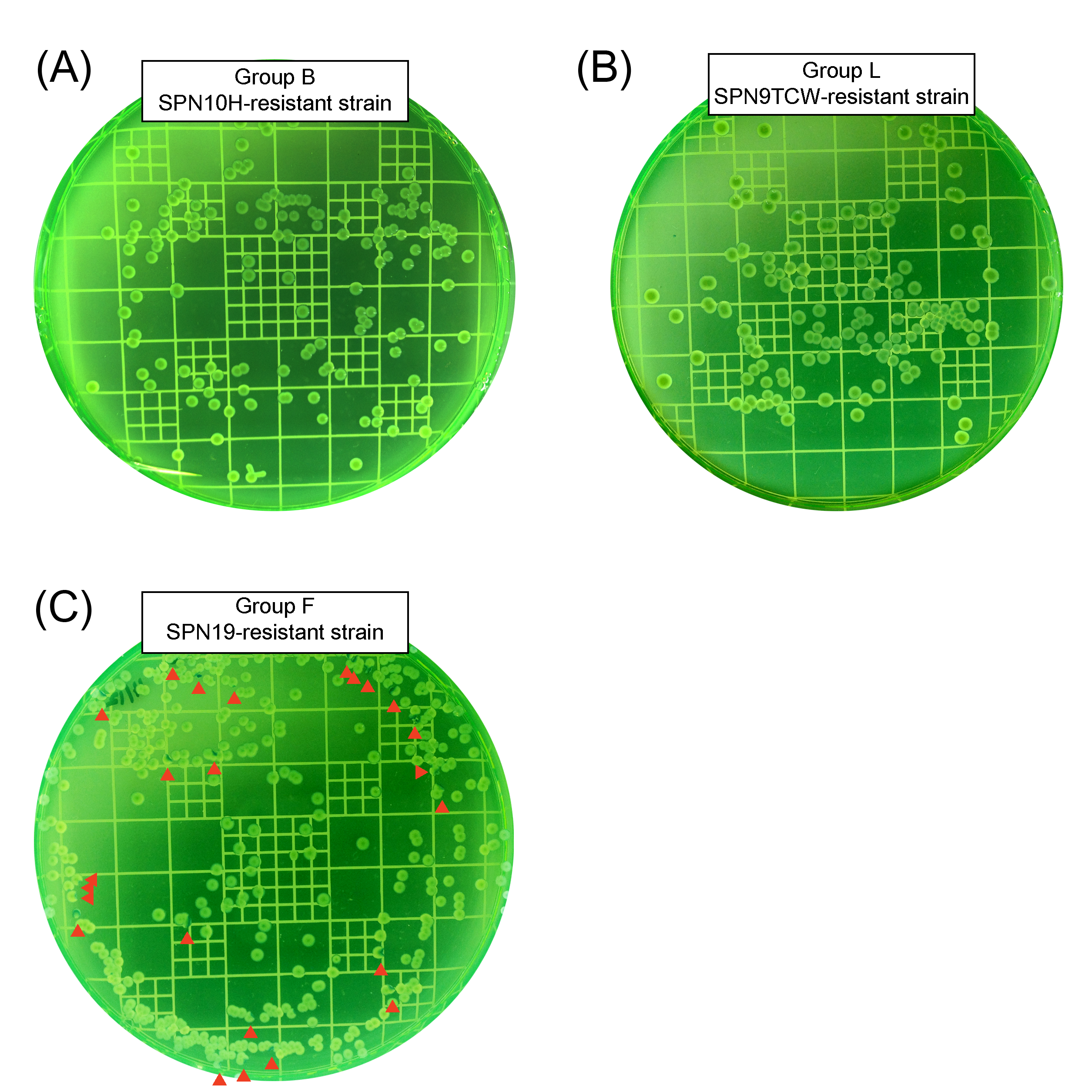

Supplement: Figure S1 — Green plate experiment of representative phages in three phage groups. (A) Group B SPN10H-resistant strain (B) Group L SPN9TCW-resistant strain (C) Group F SPN19-resistant strain. Red triangles indicate blue colonies on green plate. (TIF) [file pone.0043392.s001.tif]
