# Supplementary material for: Receptor Diversity and Host Interaction of Bacteriophages Infecting Salmonella enterica Serovar Typhimurium
Source: PLoS One. 2012 Aug 21;7(8):e43392. doi: 10.1371/journal.pone.0043392 (PMC3424200; doi:10.1371/journal.pone.0043392)
Supplement: Table S1 — Primers used in this study. (DOC) [file pone.0043392.s002.doc]

**Table S1.** Primers used in this study

| **Target** | **Primer** | **Sequence** | **Reference** |
| --- | --- | --- | --- |
| *flgK* |  |  |  |
| deletion-F | flgK-lamb-F1 | 5’-GTCCACGTAGTCGCTGCCGATAACAACGA GTATTGAAGGATTAAAAGGAACCATCTGTAGGCTGGAGCTGCTTCG-3’ | This study |
| deletion-R | flgK-lamb-R1 | 5’-CTCATATTTTGTTCGTACATCATCTGGGTA CTGATACGCATGTCATCCTTCTCCTATTCCGGGGATCCGTCGACC-3’ | This study |
| Complement-F | flgK-pACYC-F | 5’-ACGAGTATTGAAAGCTTAAAAGGAAC-3’ | This study |
| Complement-R | flgK-pACYC-R | 5’-ACTGATACGCATGCCATCCTTC-3’ | This study |
|  |  |  |  |
| *fliR* |  |  |  |
| deletion-F | fliR-lamb-F1 | 5’-ATTACGTGCGCACTCTGTTCAGCAATTTAC CTTATATCATCGGATAAACAGAACGTGTAGGCTGGAGCTGCTTCG-3’ | This study |
| deletion-R | fliR-lamb-R1 | 5’-TTTAAAATTTATTTTCGGATAAACCTTAGT AAAACAGGATAAAAATTATGGGTTAATTCCGGGGATCCGTCGACC-3’ | This study |
| Complement-F | fliR-pACYC-F | 5’-TTCAGCAATTAAGCTTATATCATCG-3’ | This study |
| Complement-R | fliR-pACYC-R | 5’-ATATCCTGGTGCATGCTTTTTAAAA-3’ | This study |
| *fliC* |  |  |  |
| deletion-F | fliC-lamb-F1 | 5’-AGCCCAATAACATCAAGTTGTAATTGATAA GGAAAAGATCTGTAGGCTGGAGCTGCTTCG-3’ | This study |
| deletion-R | fliC-lamb-R1 | 5’-CCGCACCCAGGTCAGAACGTAACGTGTCA ACCTGTGCCAAATTCCGGGGATCCGTCGACC-3’ | This study |
|  |  |  |  |
| *fljB* |  |  |  |
| deletion-F | fljB-lamb-F1 | 5’-GGTATCTCCATTGCGCAGACCACTGAAGG CGCGCTGAACGTGTAGGCTGGAGCTGCTTCG-3’ | This study |
| deletion-R | fljB-lamb-R1 | 5’-AGATCAGAGCGCAGCGCATCCACCTGCGC CAGCGCGGCATATTCCGGGGATCCGTCGACC-3’ | This study |
|  |  |  |  |
| *rfaL* |  |  |  |
| deletion-F | rfaL-lamb-F1 | 5’-CTGGTTTTTCTTTTTGTTGCCACGTATTTTC TGGATGGTATGTAGGCTGGAGCTGCTTCG-3’ | This study |
| deletion-R | rfaL-lamb-R1 | 5’-TGGATAATCGACAACGCGTTTATTATAAAC ACCATCATACATTCCGGGGATCCGTCGACC-3’ | This study |
| Complement-F | rfaL-pUHE-F | 5’-GCCACAAGCGAATTCGGAAGATT-3’ | This study |
| Complement-R | rfaL-pUHE-R | 5’- TACCGTAATAAGGATCCGCGCGTT-3’ | This study |
|  |  |  |  |
| Real-time PCR |  |  |  |
| *btuB* forward | RT_btuB_F | 5’-AGGACACTAGCCCGGATACC-3’ | This study |
| *btuB* reverse | RT_btuB_R | 5’-CAGTACATGGCTGGAGTTGG-3’ | This study |
| *fliC* forward | RT_fliC_F | 5’-CTCGGCTACTGGTCTTGGTG-3’ | This study |
| *fliC* reverse | RT_fliC_R | 5’-AGTTGCAAATGCTGATTTGA-3’ | This study |
| Control forward | RT_rrsH_F | 5’-CGGACGGGTGAGTAATGTCT-3’ | This study |
| Control reverse | RT_rrsH_R | 5’-CTCAGACCAGCTAGGGATCG-3’ | This study |
